# Preparation and Applications of Salt-Resistant Superabsorbent Poly (Acrylic Acid-Acrylamide/Fly Ash) Composite

**DOI:** 10.3390/ma12040596

**Published:** 2019-02-16

**Authors:** Wenjuan Zhu, Yagang Zhang, Penglei Wang, Zhiyong Yang, Akram Yasin, Letao Zhang

**Affiliations:** 1 Xinjiang Technical Institute of Physics and Chemistry, Chinese Academy of Sciences, Urumqi 830011, China; zhuwj@ms.xjb.ac.cn (W.Z.); wangpl@mail.dlut.edu.cn (P.W.); yangzy@ms.xjb.ac.cn (Z.Y.); akram@ms.xjb.ac.cn (A.Y.); zhanglt@ms.xjb.ac.cn (L.Z.); 2 University of Chinese Academy of Sciences, Beijing 100049, China; 3 Department of chemical and environmental engineering, Xinjiang Institute of Engineering, Urumqi 830026, China

**Keywords:** poly (acrylic acid-acrylamide), fly ash, superabsorbent, salt-resistance, Rhodamine B, adsorption

## Abstract

Solution polymerization synthesized alt-resistant superabsorbent poly (acrylic acid-acrylamide/fly ash) composites. The mass ratio of acrylic acid (AA) to acrylamide (AM), the concentration of crosslinker, the neutralization degree (ND) of AA, and the polymerization temperature were investigated by single-factor method. Optimized conditions for the synthesis of poly (acrylic acid-acrylamide/fly ash) (PAA-AM/FA) are, as following: m (AA)/m (AM) is 1.5, the content of crosslinker N, N-methylenebisacrylamide. (MBA) is 0.7%, neutralization degree of AA is 70%, polymerization temperature is 70 °C, and fly ash (FA) content is 50%. The prepared PAA-AM/FA demonstrated superior water absorption performance. The absorption capacities of PAA-AM/FA for pure water and 0.9% NaCl solution were found to be 976 g·g^−1^ and 81 g·g^−1^, respectively. Furthermore, PAA-AM/FA was found to have excellent adsorption capacity (148 mg·g^−1^) for Rhodamine B in water. Fourier Transform-Infrared Spectroscopy (FT-IR), Thermogravimetric Analysis (TGA), and Scanning Electron Microscopy (SEM) characterized the prepared materials. Results showed that fly ash was incorporated into the macromolecular polymer matrix and played a key role in improving the performance of the polymer composites.

## 1. Introduction

Fly ash, as industrial by-product of combustion of pulverized coal, is collected from coal combustion at power station and coal based chemical industry. With the development of power industry, the amount of fly ash generated increases dramatically [1]. It is reported that the quantity of coal fly ash that is generated in China has exceeds 580 million tons per year [2]. Fly ash has been one of the largest solid wastes causing environmental impacts in the form of air, water, and soil [3,4]. Therefore, the comprehensive utilization of fly ash is of paramount important for environmental protection. Studies showed that fly ash is a potential and versatile adsorbent for wastewater [5,6] and the removal of pollutants, such as dyes from aqueous solutions [7,8]. From a chemical structure point of view, fly ash is aluminosilicate layered [9,10]. Abundant hydrophilic hydroxyls are decorated on its surface, and a large number of exchangeable cations are located between interlayers, which give fly ash excellent dispersibility in aqueous solution. These features make fly ash a promising starting material for preparing organic/inorganic hybrid superabsorbent composite materials [11].

As crosslinked hydrophilic polymers, superabsorbents have been widely used in many fields, such as agriculture [12], horticulture [13], drug delivery [14], sanitary goods [15], artificial snow [16], dye removal [17], and wastewater treatment [18]. Efforts have been focused on decreasing preparation cost, improving salt tolerance, and making the material environmentally degradable. For example, starch [19], cellulose (Cell) [20], chitosan (CTS) [21], attapulgite (AT) [22], montmorillonite (MMT) [23], kaolin [24], and other natural resources were grafted and then incorporated into polymeric superabsorbent in order to improve their swelling and salt-resistant property [25,26,27,28]. Results showed that these hybrid superabsorbent resins demonstrated improved comprehensive performance, including salt tolerance, gel strength, and thermal stability. Most research on superabsorbent gels aim to achieve high absorbency for pure water, however, in real applications, the water body mostly contains a significant amount of electrolyte. Therefore, it is critically important and practically useful to enhance the absorption capacity of superabsorbent for salt-water. It would be even more ideal to utilize industrial waste, such as fly ash, to prepare a hybrid composite superabsorbent. Although the utilization of fly ash as particle enhancement for polymers has been reported, preparing value-added polymer composites with fly ash for salt-water and dye adsorption has rarely been investigated. Along this line, organic-inorganic superabsorbent composite poly (acrylic acid-acrylamide/fly ash) (PAA-AM/FA) was synthesized by aqueous solution polymerization. The effects of various factors on water absorbency in 0.9% NaCl solution were studied in detail and the adsorption capacity of PAA-AM/FA for Rhodamine B (a carcinogenic and mutagenic organic dye, which is difficult to degrade.) in water solution was also investigated. The results showed that, when 50% fly ash was added into the PAA-AM/FA composite, the composite still had good formability and elasticity, showing decent absorption for 0.9% NaCl solution and excellent adsorption to Rhodamine B solution.

## 2. Materials and Methods

### 2.1. Materials

Acrylic acid (AA), acrylamide (AM), N, N-methylenebisacrylamide (MBA), potassium persulfate (KPS), sodium hydroxide, and methanol were of analytical grade and were purchased from Tianjin Chemical Reagent Factory, Tianjin, China. AA was distilled under reduced pressure for further purification before use. AM was purified by recrystallization from benzene. KPS was recrystallized from water. MBA and other reagents were used as received. Fly ash (FA) was obtained from the power plant of China Shenhua Group Co. Ltd. (Urumqi, Xinjiang, China) It was calcined at 800 °C to decarbonize and it was milled prior to use.

### 2.2. Synthesis of PAA-AM/FA Superabsorbent Composite

PAA-AM/FA superabsorbent resin was prepared, as follows: For a typical experiment, 9.0 g AA was neutralized with 8 M sodium hydroxide solution in an ice bath under stirring. Subsequently, this neutralized AA and 6.0 g AM were added to 15 mL distilled water in 250 mL four-neck flask that was assembled with a reflux condenser, a thermometer, and a gas inlet tube. Afterwards, 0.11 g of MBA was added, the mixture was magnetically stirred for 30 min before 7.5 g of FA were added. After the FA was evenly dispersed, the mixture was gradually heated to 50 °C, while 0.10 g of KPS was added. The polymerization reaction was continued for an additional 60 min at 70 °C. The whole process was under the protection of nitrogen atmosphere. The resulting product was washed with distilled water and then dried to a constant weight in a vacuum oven at 70 °C. All of the samples were milled and screened into particles with size of 40–80 mesh for further tests. The PAA-AM/FA superabsorbent composite was obtained.

### 2.3. Measurement of Water Absorbency

The swelling capabilities of the prepared materials were measured using distilled water and saline water. For each experiment, the dried PAA-AM/FA superabsorbent composites (m_1_) was weighted and immersed into 1000 mL distilled water or 100 mL 0.9% NaCl solution at room temperature. At constant time intervals (30 min), the swollen samples were carefully weighed until it reached constant weight (m_2_). The swelling ratio Q was then calculated from the following Equation (1):

(1) $$Q=\frac{m_{2}-m_{1}}{m_{1}}$$

where m_2_ and m_1_ are the weights of the soaked gel and the dried gel, respectively. Q is calculated as grams of water per gram of dried gel. The results are shown in Table 1.

### 2.4. Measurement of Water Retention

Water retention of superabsorbent is the ability to maintain its segregation state of physical expansion after absorbing water solution, which is the opposite of dehydration ability. There are mainly two kinds of superabsorbent dehydration: heated evaporation and pressure dehydration (that are caused by pressure, centrifugal force, etc.) [29]. In practical application, the environment where the superabsorbent used is usually under pressure or high temperature. Therefore, water retention under pressure and high temperature are carried out, respectively.

#### 2.4.1. Water Retention under Pressure

Superabsorbent PAA-AM/FA composite and PAA-AM in the 0.9% NaCl solution used in the above tests were centrifuged for 10, 20, 30, and 50 min at the speed of 4000 rpm and then weighed, respectively. Water retention (R) was obtained using the following Equation (2):

(2) $$R=\frac{m_{4}}{m_{3}}\times 100\%$$

In which m_3_ and m_4_ represent the weights of the superabsorbent before and after centrifugation, respectively. The results are shown in Table 2.

#### 2.4.2. Water Retention at High Temperature

After reaching absorption equilibrium in 0.9% NaCl solution, superabsorbent PAA-AM/FA composite and PAA-AM were heated for 12 h at 75 °C in the oven and then weighed, and water retention R was also calculated according to procedure in Section 2.4.1 using Equation (2). In which m_3_ and m_4_ represent the weights of the sodden superabsorbent before and after heat treatment, respectively. The results were summarized in Table 3.

### 2.5. Characterization

The chemical composition analysis was carried out with SPECTRO XEPOS X-ray fluorescence spectrometer (XRF, Ametek- spectro Analytical Instruments Co., Ltd. Berlin, Germany) with X-LAB PRO software package (X-LabPro 2.5, Ametek- spectro Analytical Instruments Co., Ltd. Berlin, Germany). The infrared spectra of superabsorbent PAA-AM/FA composite and PAA-AM were recorded on a DIGILAB FTS 3000 Fourier Transform-Infrared Spectroscopy (FT-IR) spectrophotometer (Digilab Inc., Boston, MA, USA) with a KBr pellet in the range of 4000–400 cm^−1^. Scanning Electron Microscopy (SEM) studies were carried out on a JSM-5600 LV scanning electron microscope (Japan Electron Optics Laboratory Co. Ltd. Tokyo, Japan) after coating the sample with platinum film using an acceleration voltage of 20 kV. Thermogravimetric analysis (TGA) was carried out in a Perkin-Elmer TGA-7 analyzer PE TG/DTA 6300 instrument (Perkin-Elmer Inc., Boston, MA, USA) over a temperature range of 20–800 °C, at a nitrogen flow rate of 50 mL min^−1^, under a heating rate of 10 °C min^−1^.

## 3. Results and Discussion

### 3.1. Chemical Composition Analysis of Fly Ash

Fly ash is a fine grained dust, which is mainly composed of melted spherical vitreous particles with a smooth surface [30]. The chemical composition of fly ash in this study was analyzed with XRF and results are tabulated in Table 4.

It was found that the sum of K_2_O and Na_2_O is as high as 5.1%. This significantly decreased the amount of alkali that is needed for AA neutralization. In order to get rid of residual carbon material, the fly ash used to prepare PAA-AM/FA composite was calcined for 4 h at 800 °C before use in order to improve the water absorbency of the composite material.

### 3.2. Optimization of Polymerization Conditions

**Scheme 1:**
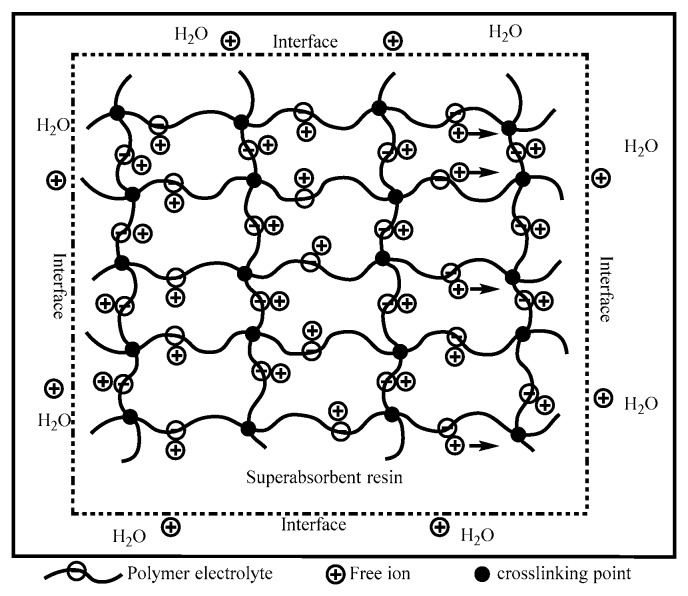
Cartoon depicting superabsorbent PAA-AM Network.

Superabsorbent composites are the three-dimensional crosslinked polymer. Their principal character is the ability to absorb and retain large volumes of water. Before expanding, the polymer chains are intertwined to form a compact gel structure; after being soaked in water, the charged groups (such as –COOH and –COO^−^) in the three-dimensional network will be solvated and dissociated to form an ionic network, as shown in Scheme 1. Charged ions in the network tend to diffuse into water due to difference of osmotic pressure. At the same time, only a small amount of ions can leave the network and enter water due to the electrostatic interaction between the positive and negative ions on the surface. Most of them still remain in the network system. The three-dimensional network of superabsorbent polymer has certain flexibility and rigidity, depending upon its environment. The effect of various parameters on the water swelling property is shown in the Flory formula [31]:

(3) $$Q^{5/3}=\frac{{\left(\frac{1}{2}\frac{i}{V_{u}}\frac{1}{S^{*1/2}}\right)}^{2}+\left(\frac{1}{2}-x\right)/V_{1}}{v/V_{0}}$$

where Q stands for the water absorbency (or swelling ratio), i/V_u_ is the charge density that is fixed on the resin before swelling, v/V_0_ is the number of effectively cross-linked chains in unit volume, S* is the ionic strength of the external solution, x is the polymer-solvent thermodynamic interaction parameter, and V_1_ is the molar volume of water. As together of $\left(\frac{1}{2}-x\right)/V_{1}$ represents the hydrophilic property of polymer electrolyte network. The term of ${\left(\frac{1}{2}\frac{i}{V_{u}}\frac{1}{S^{*1/2}}\right)}^{2}$ represents the ionic osmotic pressure and the whole numerator term stands for the swelling capacity. The crosslinking density and water absorption is inversely proportional. Therefore, a reasonable crosslinking density must be attained in order to obtain the desirable water absorbency. The effects of crosslinker concentration and other reaction conditions (reaction temperature, mass ratio of AA/AM, neutralization degree of AA, and FA concentration) on water absorbency were studied in detail. The neutralization degree of AA is relative to the mole fraction of acrylic acid monomer. The concentration of fly ash and crosslinker is the weight percent relative to both acrylic acid and acrylamide monomers.

Theoretically, with the addition of fly ash, the superabsorbent PAA-AM/FA composite can benefit from the increased ionic osmotic pressure difference and the hydrophilicity and improved salt resistance due to increased ion exchange ability.

#### 3.2.1. Effect of Polymerization Temperature on Water Absorbency of PAA-AM/FA in 0.9% NaCl Aqueous Solution

**Figure 1:**
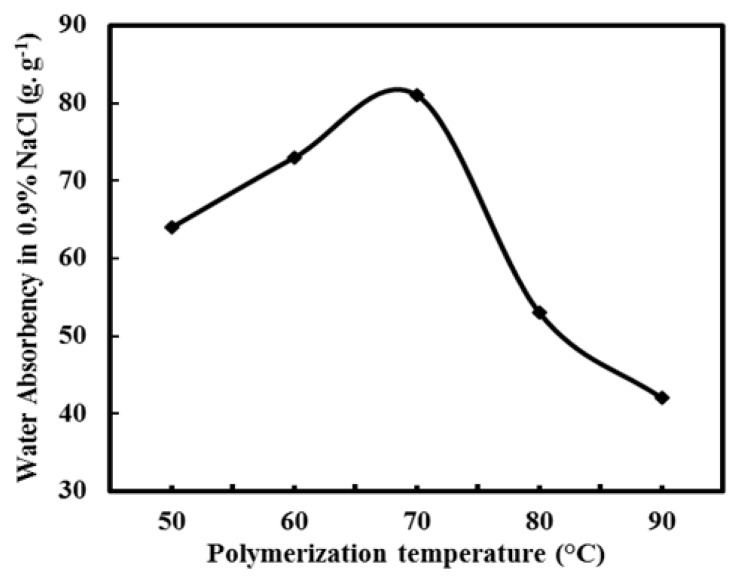
Effect of polymerization temperature on water absorbency of PAA-AM/FA in 0.9% NaCl aqueous solution (mass ratio of AA/AM, 1.5; neutralization degree of AA, 70%; crosslinker concentration, 0.7%; Fly ash (FA) concentration, 50%).

Firstly, the effect of polymerization temperature of PAA-AM/FA on its water absorbency was investigated. Figure 1 showed the relationship between the polymerization temperature and the swelling capacity of superabsorbent composite. With increasing polymerization temperature from 50 °C to 70 °C, the swelling capacity increased from 62 to 81 g·g^−1^. However, the water absorbency decreased from 81 to 41 g·g^−1^ when further increasing the polymerization temperature from 70 °C to 90 °C. It is proposed that reasonable high polymerization temperature could provide the necessary energy for the polymerization reaction to achieve quality polymeric network structure. However, it is easy to cause side reactions and decomposition of the polymer under excessive high temperature, forming chains of low molecular weight, which is disadvantageous to the absorption ability of the polymer for water. This is consistent with the reported study [27].

#### 3.2.2. Effect of Mass Ratio of AA/AM on Water Absorbency of PAA-AM/FA in 0.9% NaCl Aqueous Solution

The hydrophilic group plays an extremely important role in the water absorbency of various superabsorbent composites. There are three kinds of hydrophilic groups, including –CONH_2_, –COO^−^, and –COOH groups in PAA-AM/FA chains that exhibit different hydrophilicity. The hydrophilicity of –COOH and –COO^−^ is much stronger than that of –CONH_2_. Theoretically, the high ratio of AA/AM is beneficial and it may enhance water absorbency of PAA-AM/FA due to increased of hydrophilicity for superabsorbent composite.

**Figure 2:**
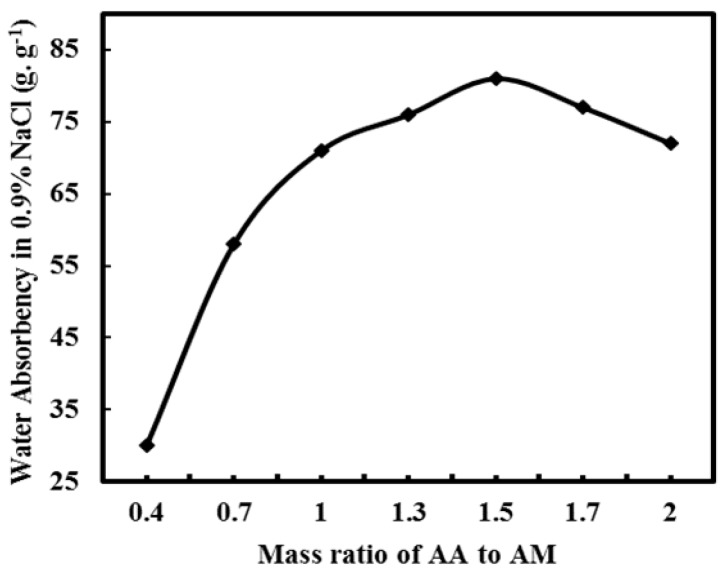
Effect of mass ratio AA/AM on water absorbency of PAA-AM/FA in 0.9% NaCl aqueous solution (polymerization temperature, 70 °C; neutralization degree of acrylic acid (AA), 70%; crosslinker concentration, 0.7%; FA concentration, 50%).

As shown in Figure 2, the water absorbency of PAA-AM/FA superabsorbent increased with increasing mass ratio of AA/AM. The optimal ratio was obtained as AA/AM = 1.5. The amide group –CONH_2_ is not easily affected by the external salt ions, which could help in enhancing the salt tolerance of the PAA-AM/FA. Therefore, the introduction of –CONH_2_ into a superabsorbent composite can enhance water absorbency in salt aqueous solution. This is consistent with the results that the water absorbency of PAA-AM/FA in 0.9% NaCl aqueous solution decreased when the mass ratio of AA/AM is large than 1.5.

#### 3.2.3. Effect of Neutralization Degree of AA on Water Absorbency of PAA-AM/FA in 0.9% NaCl Aqueous Solution

**Figure 3:**
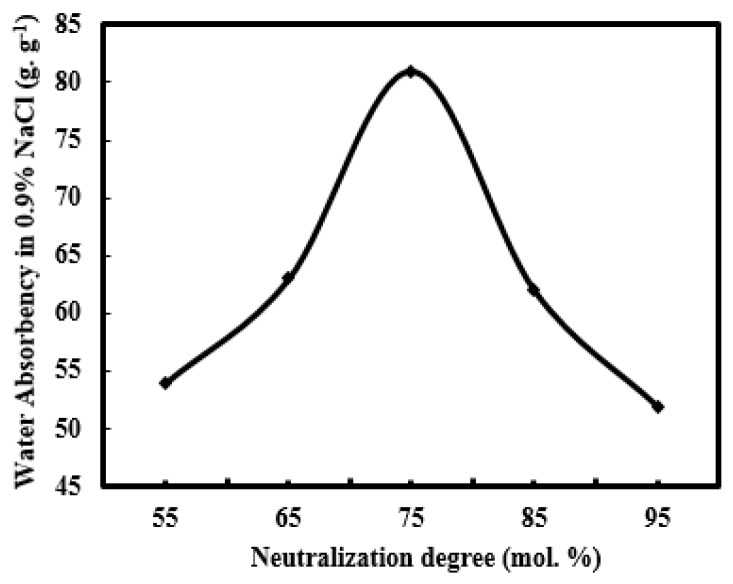
Effect of neutralization degree on water absorbency of PAA-AM/FA in 0.9% NaCl aqueous solution (polymerization temperature, 70 °C; mass ratio of AA/AM, 1.5; crosslinker concentration, 0.7%; FA concentration, 50%).

According to Equation (3), the water absorbency (or swelling ratio) at equilibrium will increase with the increasing ionic charges in the network. From another point of view, the contribution to the hydrophilicity of –COO^−^ group is much stronger than that of the –COOH group. When adding an appropriate amount of NaOH, some –COOH will be converted to –COO^−^. This is why water absorbency usually goes up with the increasing neutralization degree at an early stage. However, the further addition of alkali causes a contraction of the gel. When the neutralization degree is too high, (for instance, over 70%), a large amount of –COONa is produced in the composite. The formation of massive –COO^−^ leads to an increase in ionic strength, which will cause electrostatic screening, preventing further swelling of the gel in saline solution [32]. As shown in Figure 3, the optimized neutralization degree was found to be 70%.

#### 3.2.4. Effect of Crosslinker Concentration on Water Absorbency of PAA-AM/FA in 0.9% NaCl Aqueous Solution

**Figure 4:**
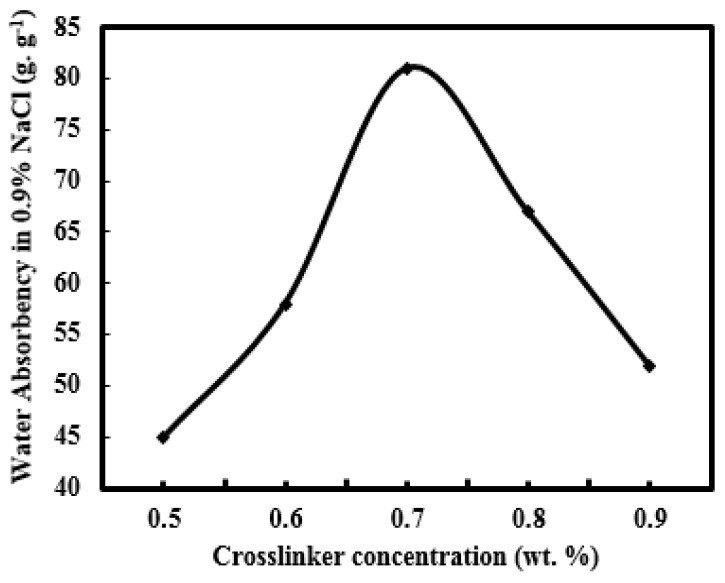
Effect of crosslinker N, N-methylenebisacrylamide (MBA) concentration on water absorbency of PAA-AM/FA in 0.9% NaCl aqueous solution (polymerization temperature, 70 °C; mass ratio of AA/AM, 1.5; neutralization degree of AA, 70%; FA concentration, 50%).

Crosslinking density is an extremely important swelling control factor. It can be seen from Equation (3) that excessive crosslinking density could decrease the elasticity of the polymer network, thus reducing the water absorbency of PAA-AM/FA in 0.9% NaCl aqueous solution. This is consistent with the experimental results in Figure 4. The water absorbency of PAA-AM/FA decreased with an increasing MBA amount in the range of 0.7–0.9% (mass percentage relative to monomer). However, water absorbency sharply increased correspondingly with the crosslinker amount in the range of 0.6−0.7%. This implied that there was a balance between the rigidity and flexibility in the PAA-AM/FA polymer network. The best crosslinker concentration of PAA-AM/FA was found to be 0.7%.

#### 3.2.5. Effect of FA Concentration on Water Absorbency of PAA-AM/FA in 0.9% NaCl Aqueous Solution

**Figure 5:**
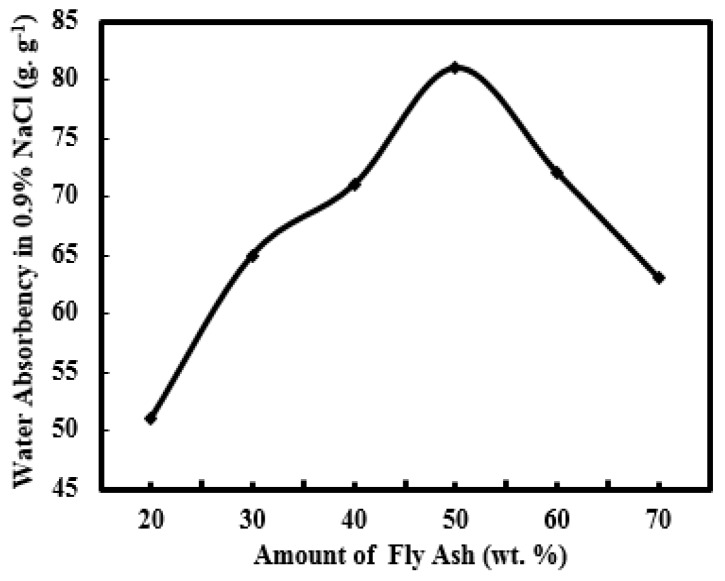
Effect of FA amount in PAA-AM/FA on water absorbency in 0.9% NaCl aqueous solution (polymerization temperature, 70 °C; mass ratio of AA/AM, 1.5; neutralization degree of AA, 70%; crosslinker concentration, 0.7%).

It was found that the water absorbency of PAA-AM/FA was dependent on the amount of FA that was incorporated into the polymer network (see Figure 5). The water absorbency of PAA-AM/FA went up with an increasing amount of FA from 20% to 50%. In the process of polymer long chain formation, solid fly ash particles were uniformly dispersed in the framework. It played a role in particle enhancement and made the polymer skeleton more stable. After swelling, more cavities and points were formed, and these nonspecific cavities and points can increase the osmotic pressure and promote the exchange of ions in polymer networks and Na^+^ in saline solution, which was why the absorbency of PAA-AM/FA in 0.9% NaCl was as high as 81 g·g^−1^, while that of PAA-AM was only 49 g·g^−1^.

**Figure 6:**
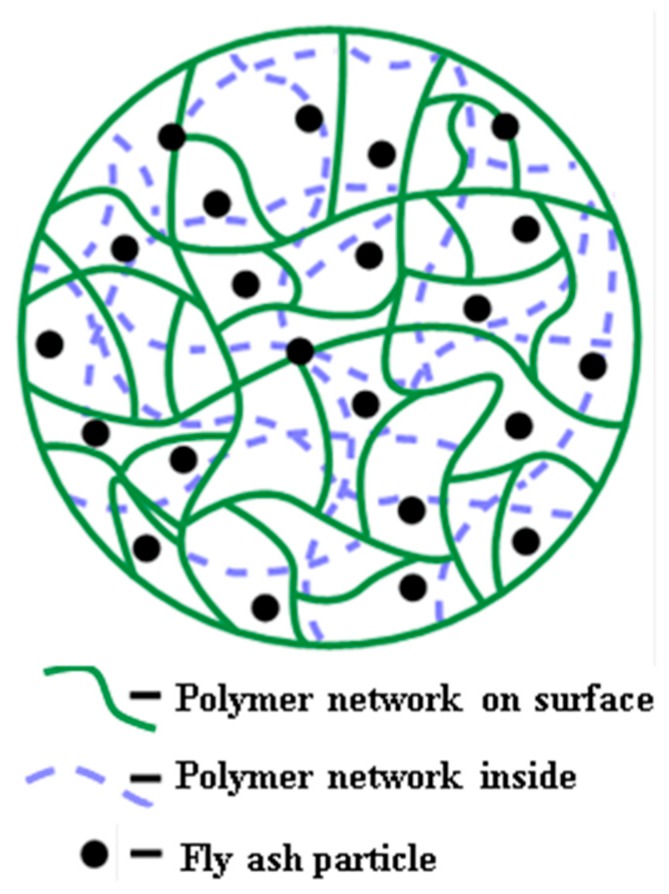
Proposed three dimensional structure of PAA-AM/FA superabsorbent composite.

It has been reported that organic-inorganic composite materials could enhance the water absorbency of superabsorbents in salt aqueous solution [33]. However, further increasing the content of FA from 50% to 70% led to a decrease in the water absorbency of PAA-AM/FA in 0.9% NaCl aqueous solution. The declining water absorbency of PAA-AM/FA with an increasing amount of FA content (over 50%) is an indication that inorganic particles are mainly physically dispersed in the polymeric network. The proposed gel network structure for the PAA-AM/FA is shown in Figure 6.

In Figure 6, the solid line represents the polymer network on the surface, and the dashed line represents the internal network structure of the polymer. By combining the solid line and the dotted line, the three-dimensional gel network structure of the superabsorbent resin is demonstrated by intertwisting the solid line and dashed line together. The results implied that an excessive amount of FA would interfere with the polymerization reaction and it was detrimental to the swelling properties of PAA-AM/FA.

### 3.3. FT-IR Spectra

**Figure 7:**
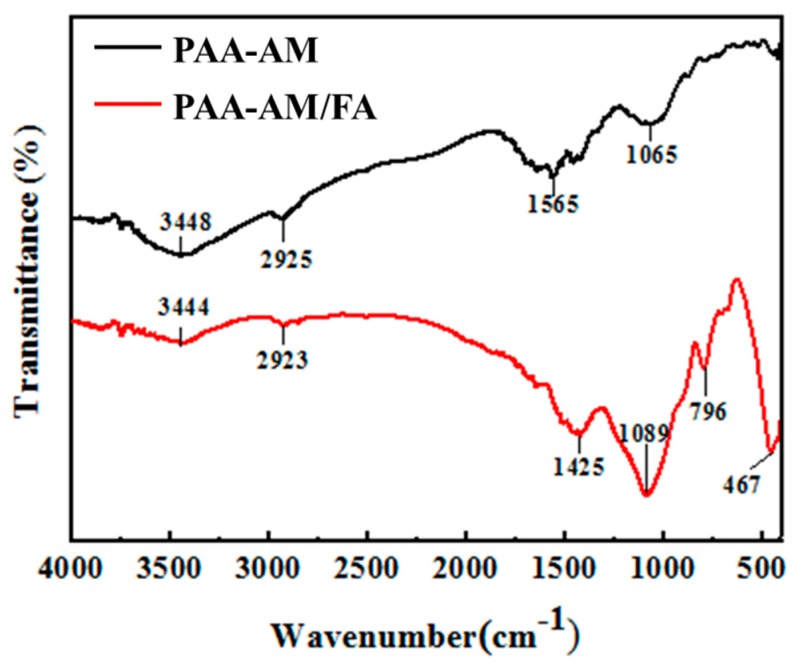
Fourier Transform Infrared Spectroscopy (FT-IR) spectra of PAA-AM/FA and PAA-AM.

As illustrated in Figure 7, the absorption band near 3448 cm^−1^ is due to the –OH from AA, the 2925 cm^−1^ is due to C–H stretching vibration, and the 1565 cm^−1^ peak is corresponding to the stretching vibration of C=O from AM. 1425 cm^−1^ is related to the symmetric stretching mode of the carboxylate anion. [11]. The –OH stretching vibration absorption peak near 3441 cm^−1^ in PAA-AM and PAA-AM/FA are both not very obvious. This is due to the reason that NaOH has neutralized the majority of the acrylic acid. When compared with the FT-IR spectrum of PAA-AM, PAA-AM/FA presents several characteristic absorption bands of FA. The 1089 cm^−1^ peak is assigned to the asymmetric stretching vibration of Si–O–Si. The 796 cm^−1^ and 467 cm^−1^ peaks are due to the symmetric stretching vibration and bending vibration of Si–O, respectively. The 2923 cm^−1^ and 467 cm^−1^ peaks are ascribed to Fe_2_O_3_ [34]. The observed changes in FT-IR spectra provide direct evidence that FA is successfully grafted onto three-dimensional PAA-AM networks.

### 3.4. Morphology Analysis

**Figure 8:**
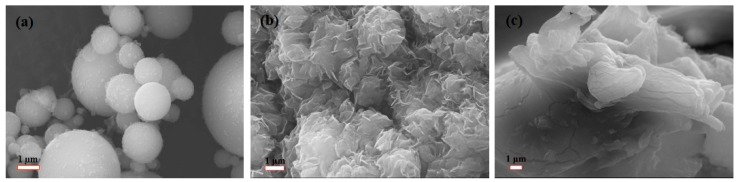
Scanning Electron Microscopy (SEM) images of FA (**a**), PAA-AM/FA (**b**), and PAA-AM (**c**).

As can be seen from Figure 8a, fly ash is a kind of semi-transparent glass microspheres whose surface is adhered to more small spherical particles. There are gaps and small holes between the ball-shaped particles that facilitate the adsorption property of fly ash [35,36]. As shown in Figure 8b, the FA particles are distributed quite uniformly in the PAA-AM macromolecule network. Noticeably, incorporating fly ash particles into matrix of sodium polyacrylate and polyacrylamide generates a unique flower shape structure with FA that was evenly wrapped by PAA-AM. There is no obvious fracture between fly ash and the polymer matrix, indicating that fly ash and neutralized PAA-AM are quite compatible. In Figure 8b, the white part is considered to be the uniform furrows of PAA-AM/FA, the gray part is the gullies in the composite, and the dark part is considered to be the cavities that formed in the reaction. When compared with the original PAA-AM polymer in Figure 8c, it can be easily observed that, after FA is introduced, there are much more furrows, cavities, and gullies on the PAA-AM/FA surface than that of PAA-AM surface. The surface morphology of PAA-AM/FA is more uniform.

### 3.5. Water Absorbency Study

The results of salt-water absorbency for superabsorbent composite with and without the addition of FA under optimized conditions are listed in Table 1. The results displayed that the salt resistance of the PAA-AM/FA polymer composite was significantly improved (from 49 g·g^−1^ to 81 g·g^−1^) as compared to PAA-AM in 0.9% NaCl solution. The prepared PAA-AM/FA polymer composite was compared with other superabsorbent composites that were reported in the literature, as well in terms of their absorbency for distilled water and 0.9% NaCl. Impressively, the absorbency for distilled water was the second highest (976 Q (g·g^−1^)), only next to the PAA-AM/MMT. While, as for the absorbency for 0.9% NaCl, to the best of our knowledge, the PAA-AM/FA composite achieved the highest value (81 Q (g·g^−1^)) among all of the superabsorbent composites that are reported in Table 5.

### 3.6. Water Retention

Table 2 and Table 3 showed the water retention capacity of soaked superabsorbent composites. When the soaked PAA-AM/FA were dried for 12 h at 75 °C in an oven, approximately 86% of the NaCl solution (0.9%) was kept, while for PAA-AM, only 73% of the NaCl (0.9%) solution was retained. Table 2 showed that the PAA-AM/FA had the water retention of 96% for 0.9% NaCl solution when centrifuged for 50 min at 4000 rpm, while for PAA-AM, only 93% of the 0.9% NaCl solution was kept under the same conditions.

### 3.7. Thermal Stability

**Figure 9:**
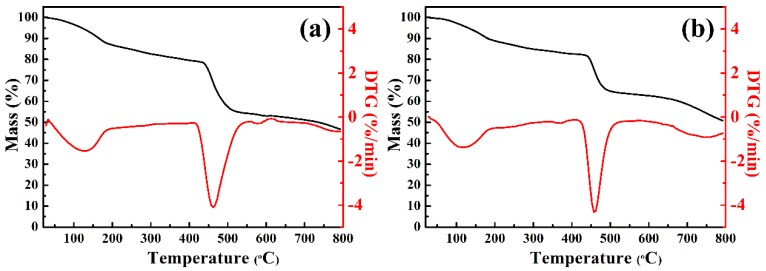
The thermogravimetric (TG) and DTG curves of PAA-AM (**a**) and PAA-AM/FA (**b**).

The stability is an important physical property for superabsorbent at elevated temperature. The thermogravimetric curves of PAA-AM and PAA-AM/FA are shown in Figure 9. It was found that, below 100 °C, two materials were both quite stable, with only small loss of weight, which was ascribed to the removal of absorbed and bonded water. The first noticeable weight loss started at 200 °C for PAA-AM/FA and 179 °C for PAA-AM. The second significant weight loss started at 462 °C for PAA-AM/FA and 459 °C for PAA-AM. These results were consistent with the work that was reported in the literature that inorganic particles can slow down the destruction of the cross-linked polymer network at an elevated temperature [45,46]. The rest of weight losses from 459 °C to 796 °C are ascribed to the breakage of main chains of polymeric backbone [47]. The thermogravimetric (TG) results suggested that the introduction of FA into composite increased the thermal stability of PAA-AM/FA.

In order to further investigate the thermal behavior of PAA-AM/FA, the DTG (Differential Thermogravimetry) measurements were carried out and the results are shown in Figure 8b. As can be seen, the maximum temperature (T_max_) of the characteristic endothermic peak is shifted from 459.1 °C (PAA-AM)) to 461.8 °C (PAA-AM/FA). Overall, the PAA-AM/FA exhibits slower weight loss and thermal decomposition rate, implying that the introduction of fly ash in superabsorbent polymer can indeed improve the thermal stability of the superabsorbent composite [48].

### 3.8. Adsorption of Rhodamine B in water by PAA-AM/FA

A batch adsorption experiment investigated the adsorption of Rhodamine B solution (initial concentration 100 mg·L^−1^) on PAA-AM/FA. The same amount of 100 mg·L^−1^ Rhodamine B solutions were placed in four test tubes marked (a), (b), (c) and (d), in which 0.10 g of PAA-AM, FA, and PAA-AM/FA were added into tubes (b), (c) and (d). After the equilibrium, the removal efficiency of Rhodamine B was found to be 15.7%, 5.3%, and 100%, respectively. The maximum adsorption capacities were calculated as 7.50 mg·g^−1^, 2.60 mg·g^−1^ and 148 mg·g^−1^ for PAA-AM, FA, and PAA-AM/FA, respectively, at room temperature (See Table 6).

**Figure 10:**
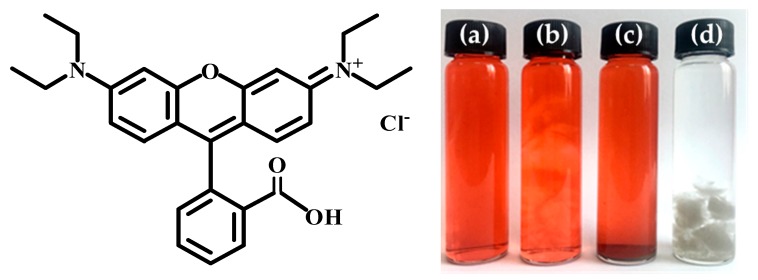
Structure of Rhodamine B and comparison of Rhodamine B adsorption and removal in water by PAA-AM, FA and PAA-AM/FA in 2 minutes. (**a**) 100 mg·L^−1^ Rhodamine B solution. (**b**), (**c**), and (**d**) are 100 mg·L^−1^ Rhodamine B solution after adding 100 mg PAA-AM, FA, and PAA-AM/FA, respectively.

It can be seen from Figure 10 that, after adding PAA-AM/FA, the Rhodamine B solution went from vividly bright red to colorless in two minutes. In contrast, adding PAA-AM or just fly ash powder into Rhodamine B solution showed no obvious color change and only achieved limited adsorption capacity. Results showed that PAA-AM/FA could be promising potential functional material for the adsorption and removal of dye Rhodamine B due to the synergistic action of polymer matrix and fly ash.

After swelling, deformation, and adsorption, the adsorption sites on the PAA-AM polymer system is not strong enough and reversible desorption is easy to occur, so the adsorption of dye is limited. When fly ash is uniformly dispersed into the PAA-AM polymer system as granular filler, the entire PAA-AM/FA polymer network becomes enhanced and more strongly fixed, enabling the adsorption sites of the original polymer network to become more stable. After swelling, the dye molecules can be more firmly grasped, so it is not easy to fall off. Meanwhile, after PAA-AM/FA swelling, the cavities and active sites that were formed by fly ash particles solidified in the polymer network greatly increase the number of adsorption points. Nevertheless, PAA-AM has no such benefits without adding FA into the polymer network. Therefore, the adsorption ability of Rhodamine B on PAA-AM/FA is much higher when compared with that using fly ash or PAA-AM alone. PAA-AM/FA prepared in this study showed outstanding adsorption performance among all of the superabsorbent composites reported in the literature in Table 7.

In this study, the concentration of Rhodamine B after being processed with PAA-AM/FA in different time was measured. The results are shown in Table 8, in which PAA-AM/FA showed a fast adsorption rate. It was found that PAA-AM/FA was effective in removing Rhodamine B from its aqueous solution. The full adsorption was completed in 10 min.

### 3.9. Preparation of Hybrid Organic-Inorganic Composite PAA-AM/FA

**Scheme 2:**
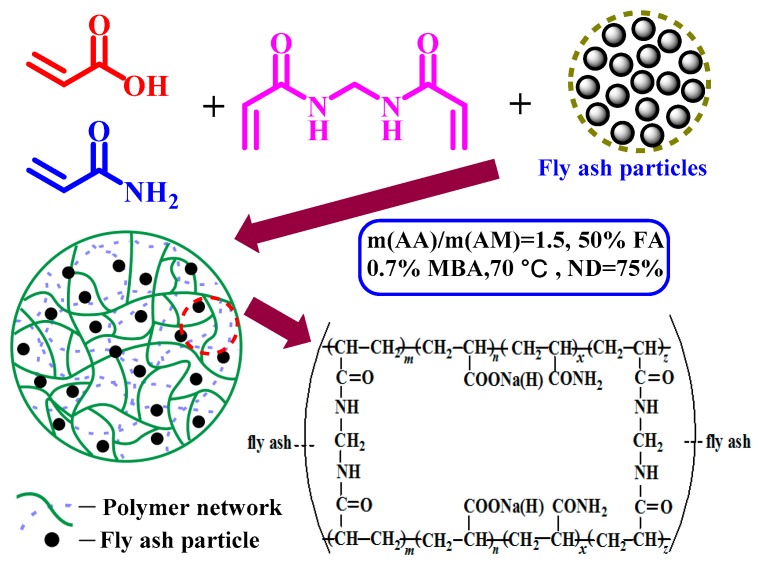
Proposed preparation process for the PAA-AM/FA composite.

Several studies have discussed the mechanism of reaction between inorganic materials and polymer matrix [49]. In this work, inorganic particles (FA particles) are proposed mainly physically dispersed into the polymeric network. An appropriate amount of FA particles can improve the thermal stability of polymeric network and improve saline water absorbency by enhanced salt resistance ability via ion exchange. In a Rhodamine B adsorption study, an interesting synergic effect was observed for the PAA-AM/FA composite. Schematically, the synthetic pathway for PAA-AM/FA composite is shown in Scheme 2.

## 4. Conclusions

Salt-resistant superabsorbent poly (acrylic acid-acrylamide/fly ash) composites were designed and synthesized by solution polymerization. The PAA-AM/FA exhibited slower weight loss and thermal decomposition rate. The prepared PAA-AM/FA demonstrated superior salt-water absorption performance due to the synergistic action of polymer matrix and fly ash. The adsorption capacity of PAA-AM/FA for pure water and 0.9% NaCl solution were found to be as high as 976 g·g^−1^ and 81 g·g^−1^, respectively. Furthermore, PAA-AM/FA demonstrated excellent adsorption capacity for Rhodamine B (148 mg·g^−1^) in water. Fly ash, as industrial waste of combustion of pulverized coal, can be potentially utilized to prepare hybrid composite superabsorbent polymers. Noticeably, the adsorption capacity of PAA-AM/FA for Rhodamine B dye was considerably higher than that of using PAA-AM and FA alone. It can be concluded that PAA-AM/FA could be a promising potential functional materials for salt-water treatment and adsorption and removal of Rhodamine B type dye.

## Figures and Tables

**Table 1:** The absorbency of superabsorbent poly (acrylic acid-acrylamide/fly ash) (PAA-AM/FA) composite and PAA-AM.

Superabsorbent Composite	Q (g·g^−1^)	
	Distilled Water	0.9% NaCl
PAA-AM/FA	976	81
PAA-AM	1019	49

**Table 2:** The water retention of PAA-AM/FA composite and PAA-AM under pressure of 4000 rpm (wt. %).

Superabsorbent Composites	Centrifuging Time (min)			
	10	20	30	50
PAA-AM/FA	99%	98%	97%	96%
PAA-AM	98%	96%	94%	93%

**Table 3:** The water retention of PAA-AM/FA composite and PAA-AM at 75 °C (wt. %).

Superabsorbent Composites	Heating Time (h)					
	1	3	5	7	9	11
PAA-AM/FA	99%	97%	95%	92%	89%	86%
PAA-AM	96%	94%	91%	87%	80%	73%

**Table 4:** Chemical composition of the fly ash used to prepare PAA-AM/FA composite using X-ray fluorescence spectrometer (XRF).

Compound	Na_2_O	K_2_O	Al_2_O_3_	SiO_2_	P_2_O_5_	SO_3_	CaO	TiO_2_	MgO	Fe_2_O_3_
**Mass (wt. %)**	3.045	2.108	21.82	52.88	0.220	0.827	8.08	0.86	1.929	5.62

**Table 5:** The absorbency for water and salt-water of different superabsorbent composites.

Superabsorbent Composite	Q (g·g^−1^)		Reference
	Distilled Water	0.9% NaCl	
PAA-AM/MMT	1024	56	[31]
PAA/SH/CTS	183	41	[37]
PAA/CTS/Cell	390	40	[38]
PAM/CMC/CTS	500	58	[39]
PAA-AM/NaAlg/RHA	830	36	[40]
PULL/PVA/MMT	143	40	[41]
PCMC/AC	142.4	69	[42]
PAA/KF/Cell	422	49	[43]
PAMPS-AM/PGS	652.6	69	[44]
PAA-AM	1019	49	This work
PAA-AM/FA	976	81	This work

Abbreviations in Table 5: SH—Sodium humate; CMC-Carboxymethylcellulose; RHA—Rice husk ash; PULL—Pullulan; AC—Activated carbon; KF—Potassium fulvate; PAMPS—poly(2-acrylamido-2-methyl-1-propanesulfonic acid).

**Table 6:** The adsorption capacity for Rhodamine B by different superabsorbent composite (mg·g^−1^).

PAA-AM/FA	PAA-AM	FA
148	7.50	2.60

**Table 7:** The adsorption to Rhodamine B by different superabsorbent composites.

Superabsorbent Composite	Dye Feed Concentration (mg·L^−1^)	Adsorption Performance mg·g^−1^ Resin	Reference
PHEMA-GMA	1000	76.8	[24]
PAA-VP	40	4.1	[40]
PAA-AM	50	6.4	[42]
Jute stick	50	4.6	[44]
PAM-HEMA	0.25–3	0.15	[45]
PAM-HEMA	500	257	[45]
PAA-AM/FA	100	148	This work
PAA-AM/FA	500	366	This work
PAA-AM	50	7.5	This work

Abbreviations in Table 7: HEMA—2-Hydroxyethyl methacrylate; GMA—Glycidyl methacrylate; VP—Vinyl pyridine.

**Table 8:** The concentration of Rhodamine B after being processed with PAA-AM/FA in different time.

Concentration (mg·L^−1^)	PAA-AM	FA	PAA-AM/FA
Initial Conc.	100	100	100
2 min later	89.0	95.8	1.90
10 min later	85.0	95.1	0
1 h later	84.3	94.7	0
